# Full‐scale modeling of chemical experiments

**DOI:** 10.1002/smo.20230010

**Published:** 2023-10-30

**Authors:** Junfeng Wang, Guohui Li

**Affiliations:** ^1^ Laboratory of Molecular Modeling and Design State Key Laboratory of Molecular Reaction Dynamics Dalian Institute of Chemical Physics Chinese Academy of Sciences Dalian China; ^2^ School of Physics Liaoning University Shenyang China

**Keywords:** artificial intelligence, full‐scale modeling, molecular dynamics

## Abstract

Computational chemistry methods are playing an increasingly pivotal role in chemical experiments. From quantum chemistry simulations to finite element simulations, researchers can always find an appropriate simulation method to elucidate the specific mechanisms at a certain resolution scale. However, in organic or inorganic synthesis, the synthesis mechanisms span multiple spatial and temporal scales of chemical experiments. Furthermore, the intricate nature of these mechanisms renders it impossible for any single simulation method to provide a comprehensive depiction of the entire process. In this perspective, using zeolite and polymer synthesis simulations as examples, we stress the significance of full‐scale modeling techniques for chemical experiments and urge the corresponding sophisticated simulation platform.

## Graphical abstract

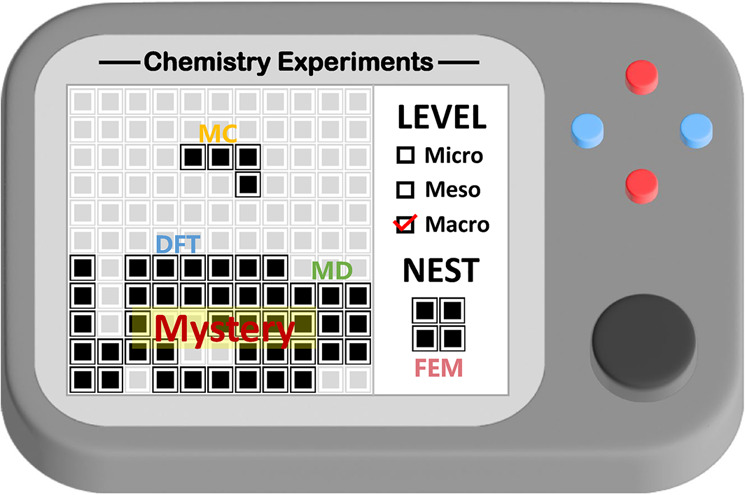

## 1 INTRODUCTION

**FIGURE 1:**
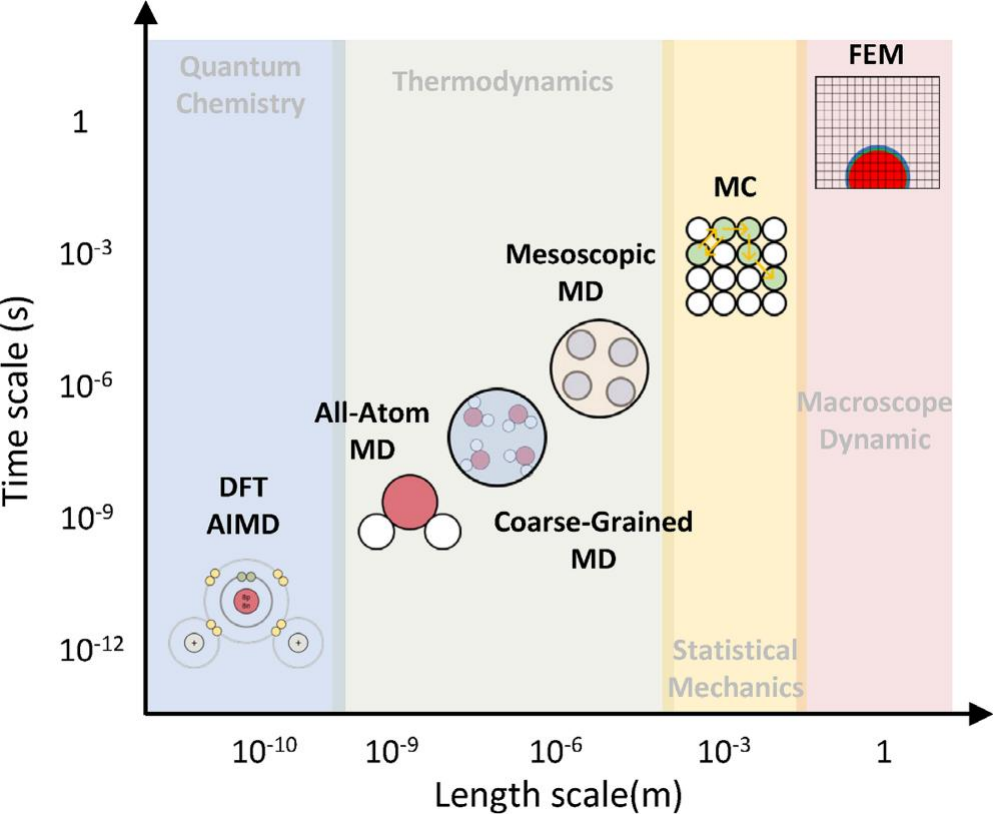
The diagram of the simulation methods at different resolution scales.

Nowadays, computational chemistry methods have become powerful tools to study the microscope process and the thermodynamic mechanism in chemical experiments, such as the self‐assembly of amphiphilic molecules, the catalytic reaction, and the molecular synthesis. Various simulation methods are rapidly developing with different resolution scales based on different theories. As shown in Figure 1, the density functional theory simulation (DFT) and the ab initio molecular dynamics (AIMD) based on quantum chemistry can show detailed information at the electronic scale. These methods are widely used in the study of chemical reaction mechanisms.[^1^, ^2^] The molecular dynamic simulations (MD) can show the microscope structures and kinetic information based on thermodynamics. These methods can sample on a larger scale to explore the molecular structures and the microscope mechanisms of diffusion, adsorption, and phase separation.^[3]^ MD simulations can be subdivided into all‐atom MD, coarse‐grained MD, and mesoscopic simulations, in which the dissipative particle dynamics simulation (DPD) is commonly used in mesoscopic simulations.[^4^, ^5^, ^6^] Monto Carlo simulations (MC) can sample at a larger spatial‐temporal scale than MD methods based on statistical mechanics, which can even solve the macrodynamic problem.^[7]^ The finite element method (FEM) can solve the mechanical properties and dynamic behavior based on the macroscope dynamic theories.^[8]^ Various theoretical tools can help people to capture more microscope details of chemical experiments, thereby enhancing the capabilities in the molecular design.

However, chemistry experiments, particularly organic or inorganic synthesis, involve a set of complex processes with multiple resolution scales, such as chemical reactions, diffusion, aggregation, and phase separation. Therefore, it is challenging to capture the complete picture of the synthesis mechanism using a single model. Consequently, most simulation works fail to provide practical guidance for optimizing synthetic systems, and there is an urgent need to develop efficient full‐scale models for chemical experiments.

In this perspective, the zeolite and polymer synthesis systems are used as examples to introduce different application scenarios of the simulation methods with different resolution scales. Then, the recent development of multiscale modeling methods is introduced. Finally, we call for further development of an efficient full‐scale modeling method to efficiently integrate various simulation methods with different resolution scales.

## 2 DISCUSSION AND PERSPECTIVE

### 2.1 The simulation studies in zeolite synthesis at different scales

The common raw materials of zeolites are aluminosilicate, and the inorganic and/or organic cations act as the directing agents for controlling cage construction. The zeolite synthesis process has three main stages, and each stage has been studied by the appropriate simulation method.

#### Reaction

**FIGURE 2:**
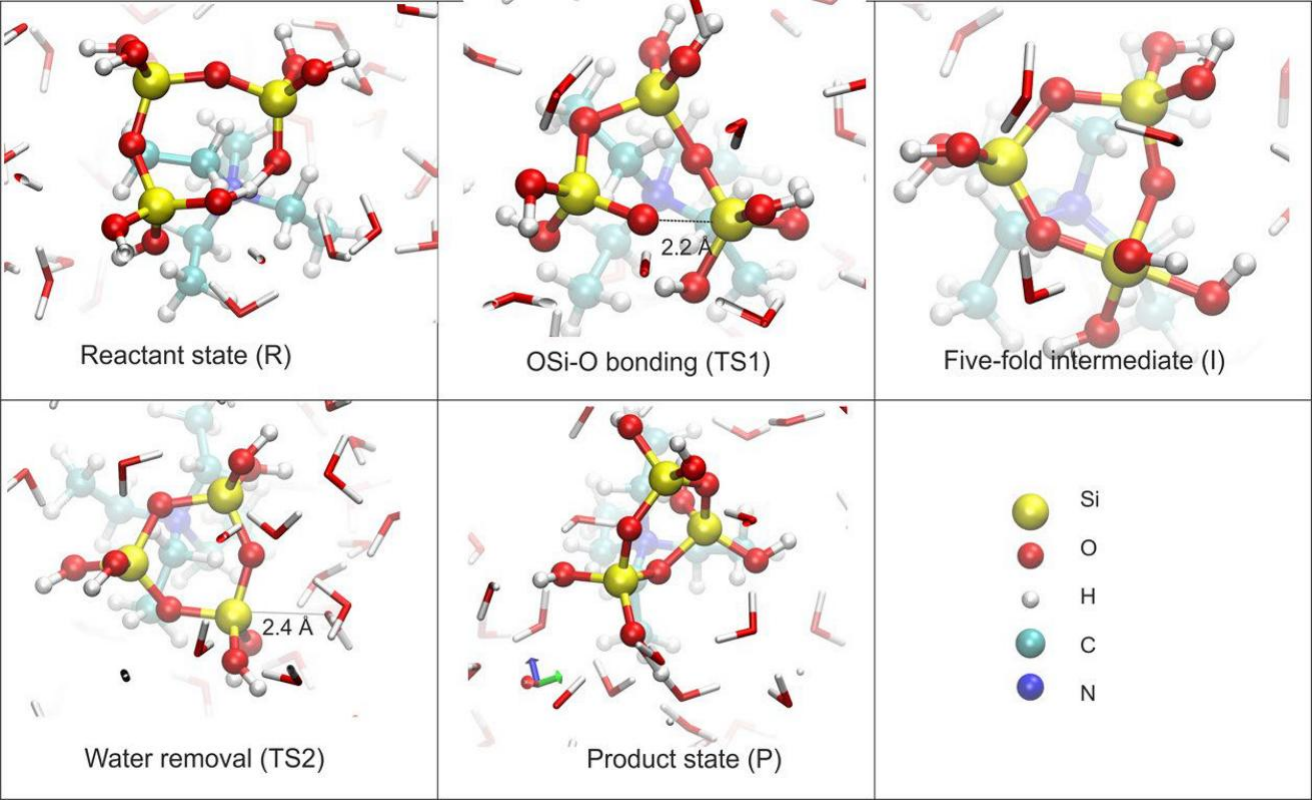
The typic intermediate states in the 3‐ring closure reaction by ab initio molecular dynamics (AIMD). Reprinted with permission from Ref. [9].

The early stage involved the condensation of aluminosilicates and the dissociation of zeolite oligomers. Trinh et al. found that the organic structure‐directing agents (OSDAs) (Tetraethylammonium) favored the 3‐ring and double 3‐ring structure of silicate oligomers by AIMD,^[9]^ and Figure 2 shows the typic intermediate states during the formation of the 3‐ring structure. Draghi et al. also investigated the impact of the OSDAs on the structural selectivity of the zeolite oligomers by combining DFT, Brønsted‐Evans‐Polanyi relationships and kinetic Monte Carlo simulations (KMC), and they found that the OSDAs could produce a higher concentration of 4‐ring oligomers.^[10]^ Prasad et al. also studied the effects of the divalent cation on the reaction pathway of dimerization by the DFT.^[11]^ In addition, to the dissociation of zeolite oligomers, Dupuis et al. discussed the complex mechanism of decondensation that involved two water molecules using MD with the reactive force field (ReaxFF), while most previous studies considered the effect of one water molecule.^[12]^

#### Mesophase

The next stage involved the formation of amorphous clusters or nanoparticles. Dupuis et al. used MD with ReaxFF to study the formation process of amorphous clusters, and they demonstrated that the aluminates served as the nucleation center using the aggregation process.^[13]^ To observe more kinetic information, it is necessary to construct a larger simulation system. Considering the computational consumption, various coarse‐grained silica models have been developed.[^14^, ^15^] For example, Jorge et al. observed the evolution process from silica polymerization to the formation of clusters using a reactive coarse‐grained silica model.^[15]^ Besides MD, Malani et al. simulated the formation of amorphous clusters by KMC at a larger space and time scales than MD, and the formation and breaking of the ring structure and the effect of solvent reactivity and precursor concentration on the porosity evolution of amorphous clusters were discussed in detail.[^16^, ^17^]

#### Crystallization

**FIGURE 3:**
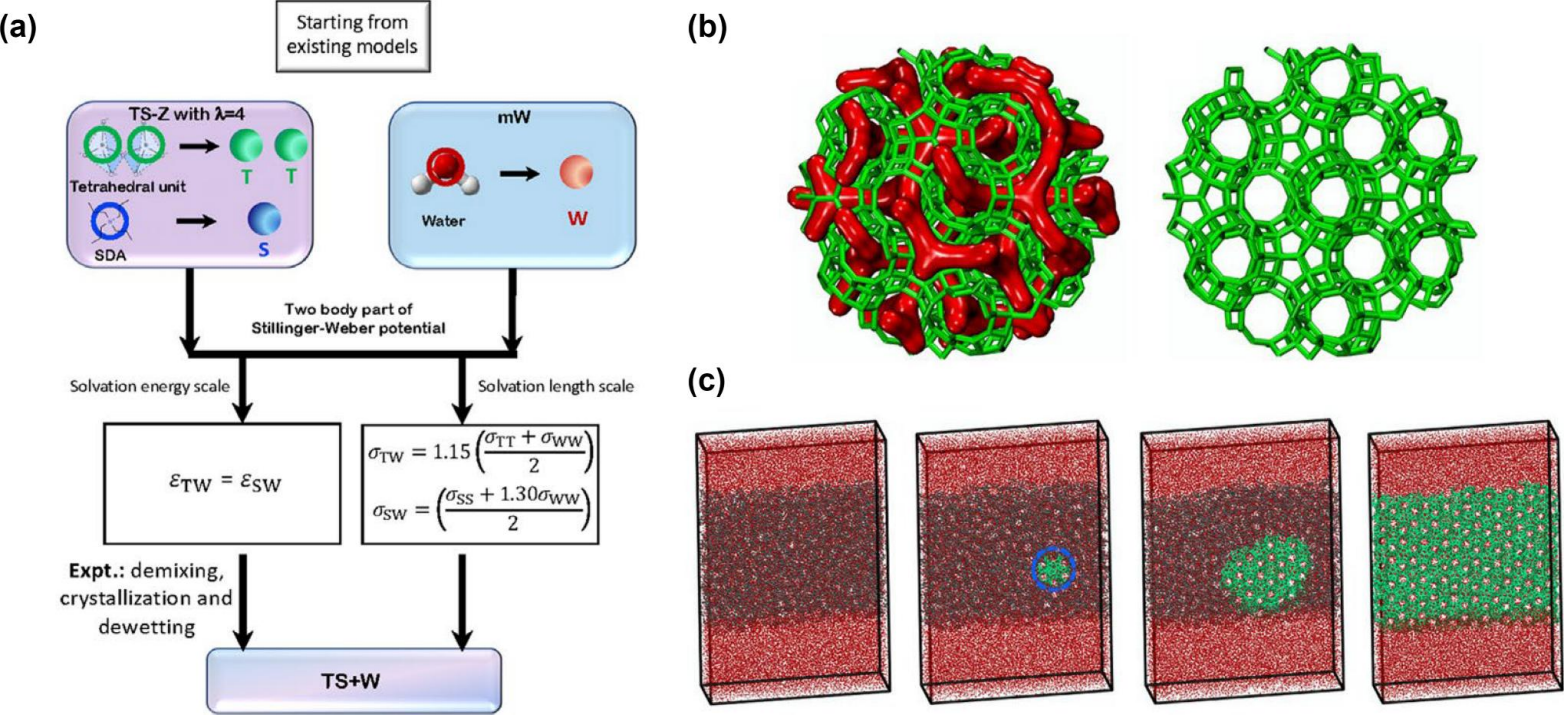
(a) Scheme of the coarse‐grained modeling method of zeolites based on the coarse‐grained water model. (b) Snapshots of zeolite Z1. (Green bonds represent silica‐like T particles and accessible channels shown with a red surface.). (c) The crystallization process of zeolite Z1 in the hydrated dense amorphous silica phase. Reprinted with permission from Ref. [18]. Copyright 2021 American Chemical Society.

In the final stage, the nucleation and crystallization processes are focused on. Molinero et al. proposed a coarse‐grained model based on the coarse‐grained water crystallization model to simulate the nucleation and crystallization of zeolites, as shown in Figure 3.^[18]^ Using the above model, they reported a series of researches on zeolites' nucleation and crystallization dynamics process. For example, they described the unclassical nucleation process, which focuses on the effect of the stability of interphase, and they also confirmed the critical nucleus size.[^19^, ^20^, ^21^, ^22^] Even though people can observe the crystallization process in tens of nanometers and several microseconds by using the coarse‐grained model, there are still limitations to the above models because they cannot describe the polymerization process of aluminosilicates. For more macroscopic crystal structure evolution, Trueman studied the transformation of crystal grain structure by KMC, which agrees with experimental results.^[23]^

Compared with the kinetic mechanism, studying the structure properties of the microscope cages can reduce the complexity of modeling. For example, Zheng et al. used AIMD to identify that the Löwenstein rule is obeyed, and the −Al−O−Al− linkage is avoided in the zeolitesynthesis.^[24]^ Even though the simulation of the zeolite synthesis process can show more dynamic details, it is difficulty to well sample a certain character. Therefore, the simulation of static systems still holds its advantages, particularly in light of the rapid advancements in machine learning methodologies. For instance, Bombarelli et al. first constructed a complete set of high‐throughput simulation frameworks, including the criteria for screening OSDAs, automatic acquisition of data from the experimental literature, automatic dynamics simulation, and construction of the database, and then new OSDAs were designed by combining the machine learning method; finally, the results of the experiments validated the efficiency of the design.[^25^, ^26^, ^27^, ^28^, ^29^]

### 2.2 The simulation studies in polymer synthesis at different scales

The polymer synthesis system is as complex as the zeolite system. Theoretical works have focused on polymer polymerization reaction and physicochemical properties in polymer synthesis.

#### Polymerization

M. Davletbaeva et al. clarified the mechanism of the high cis‐stereospecificity of 1,3‐butadiene during the polymerization in the neodymium‐based Ziegler–Natta system by DFT and the onion method.^[30]^ In addition, to the familiar polymerization reactions, some reaction models are combined with MD to obtain more kinetic information at a larger spatial and temporal scale. For instance, R. Gissinger et al. developed a reaction model for the atomistic MD and showed the condensation of nylon‐6,6 and the addition reaction of styrene.^[31]^ To the coarse‐grained MD, a simpler model, the chain end growth model, is proposed to reflect the polymerization process of polymers. Zhang et al. used the above model to study how the dispersity of molecular mass affects the diffusion coefficient, glass transition, and fragility of polymers prepared by reversible addition‐fragmentation chain transfer (RAFT) polymerization.^[32]^ MC is also an efficient method to study the polymerization reaction process. Nagaoka et al. used a hybrid Monte Carlo/molecular dynamics method, the Red Moon (RM) method, to study the 1‐octene polymerization reaction catalyzed by the ionic pair at different monomer concentrations, where the degree of polymerization was consistent with experimental results.^[33]^ Matyjaszewski et al. studied the growth and crosslinking of polymers in a confined space and clarified the influence rule of the space width, grafting density, and initial initiator/crosslinker ratios on the gel point.^[34]^ Shen et al. described the effect of the heterogeneous reaction environment on surface‐initiated polymerization and found that the different chain growth environments lead to a large dispersity and a broader molecular weight distribution.^[35]^ Even though using the MC method, it still requires a huge computational cost to describe monomer conversion and macromolecular quantities accurately. Therefore, various accelerated MC methods are proposed. For example, Broadbelt et al. proposed a novel approach for accelerating KMC simulations by a factor of ∼100 through scaling relationships.^[36]^ Chen et al. combined the multistep method and the “buffer pool” concept with the steady‐state MC to accelerate the simulation of the polymerization process.^[37]^ In addition, it is worth noting that the degradation of polymers, the opposite of polymerization, is also essential, which has been studied by MD or the moment method.^[38]^

#### Physicochemical properties

**FIGURE 4:**
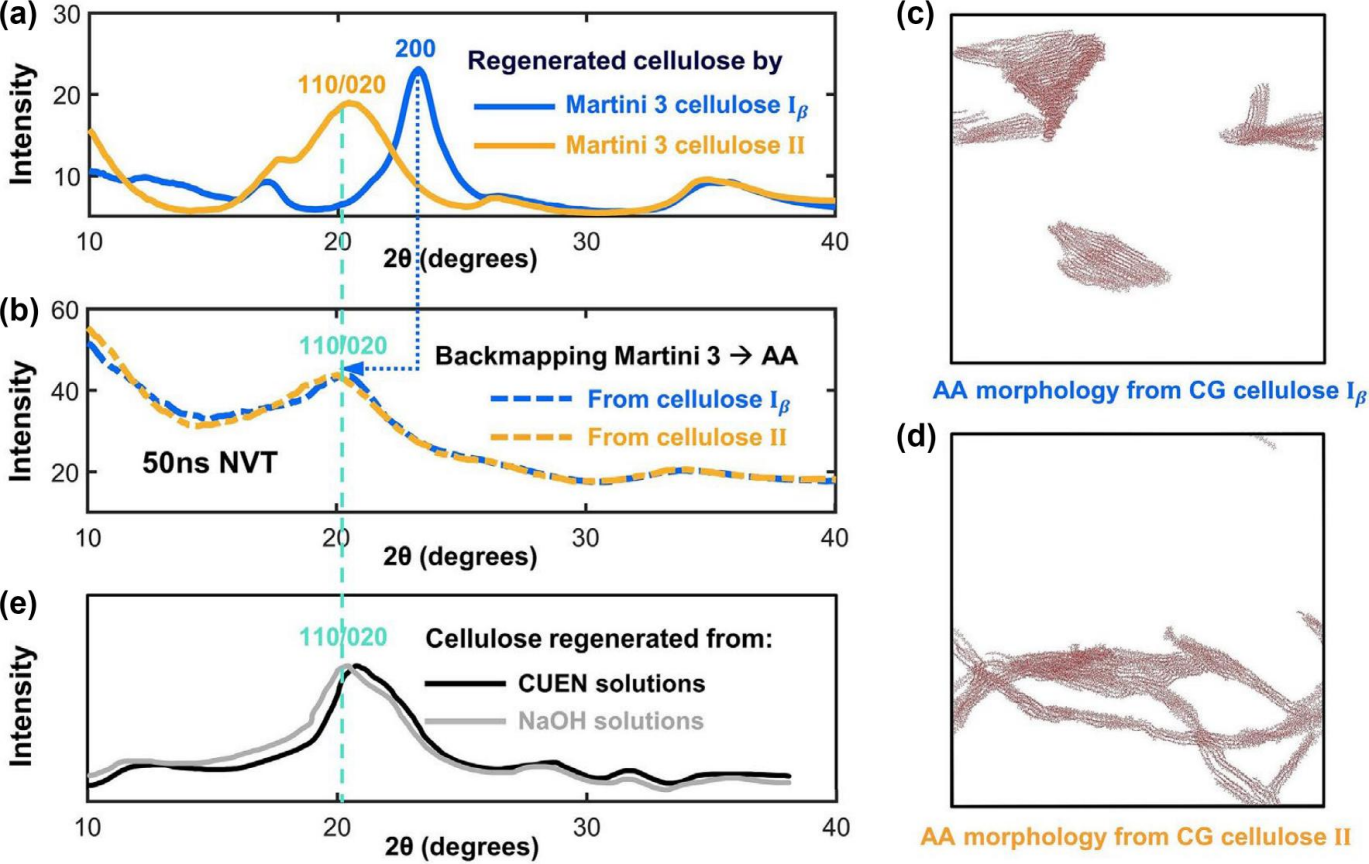
(a) The simulation X‐Ray diffraction (XRD) curves of two cellulose structures obtained by the coarse‐grained molecular dynamic simulations (MD). (b) The simulation XRD curves of the cellulose structure regenerated by all‐atom MD, and the all‐atom initial model is backmapped from coarse‐grained MD. (c) and (d) The final all‐atom morphology backmapped from the coarse‐grained morphology. (e) The experimental XRD curves of cellulose structures in the different solutions. Reprinted with permission from Ref. [39].

The phase separation of polymers is critical for their physicochemical properties. For instance, Zozoulenko et al. first only used all‐atom MD to study the regeneration process of cellulose, and they could not observe the order structure. Then, they used the coarse‐grained and all‐atom MD to study the regeneration process of cellulose. As shown in Figure 4, by backmapping the coarse‐grained morphology to atomic models, they reproduce the cellulose regeneration structure, which is in good agreement with experimental results.[^39^, ^40^] Zhang et al. also used the coarse‐grained MD to study the effects of linear or cyclic polyethylene entanglement on the crystal nucleation.^[41]^ With the rapid development of machine learning technology, people can summarize the properties of various polymers and forecast the properties of unknown polymers. For instance, Farimani et al. constructed a prediction model for the polymer properties based on the Transformer‐based language model.^[42]^ Luo et al. designed new polymers with high thermal conductivity by combining the Bayesian neural network (BNN) and MD.^[43]^

### 2.3 Research progress of the multi‐scale modeling method

As our above examples show, whether organic or inorganic systems, the difficulty of simulations for chemical synthesis experiments lies in the large space‐time span involved in the synthesis mechanism. A sequence of synthesis events occurring at different resolution scales are always interdependent, such as the chemical reaction, diffusion, aggregation, and adsorption. Indeed, it is still challenging to clear the whole picture of the synthesis mechanism. However, most traditional simulation works are limited to the single resolution scale, which is unable to provide effective improvement schemes for chemical experiments. To solve this problem, it's necessary to develop full‐scale modeling methods. Currently, there have already been three potential development directions.

#### Enhanced sampling method

The method's core idea is that the low‐resolution simulation method is used for sampling, and the high‐resolution simulation method provides the screening criteria for sampling or fine structures. This method has been successfully used in the evolution of ring structure in zeolite synthesis.^[10]^ Besides that, Streitz et al. bridged the Continuum, coarse‐grained, and all‐atom scales by the machine learning method to describe the protein‐lipid interaction from the μm to nm scale.^[44]^ Archontis et al. used MC to rapidly sample side‐chain mutations and MD to refine the molecular structure for protein design.^[45]^

#### Onion method

**FIGURE 5:**
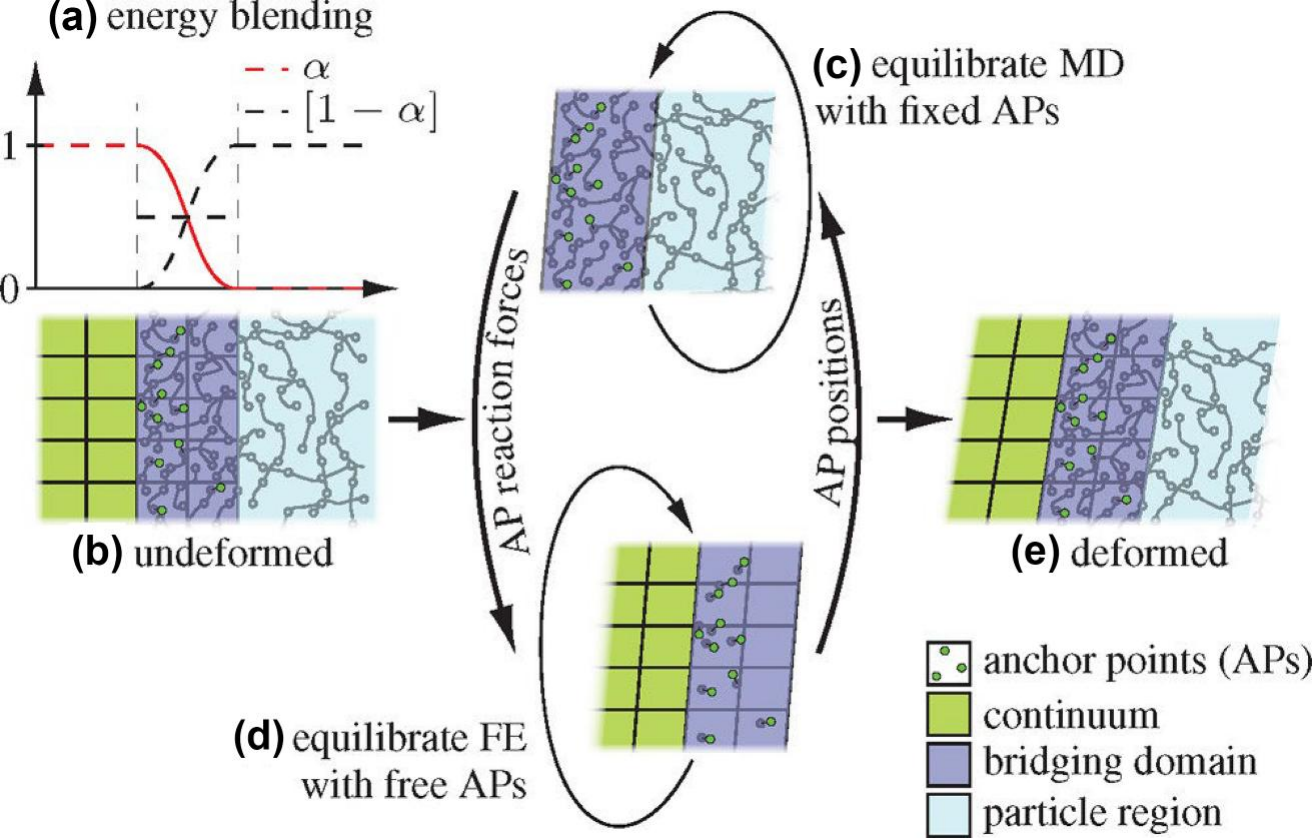
The diagram of molecular dynamic simulations (MD) and finite element method (FEM)‐coupled solution using the Capriccio method: The bridging domain is the junctional zone between MD and FEM, and a constant or cubic energy weighting is employed (a); the undeformed configuration (b) is iteratively equilibrated with MD (c) and FEM (d) until the deformed configuration in equilibrium (e) is reached. Reprinted with permission from Ref. [49]. Copyright 2022 American Chemical Society.

This method is the most direct and earliest multiscale simulation method to integrate different resolution simulation methods into one system. The onion method first chooses the central domain, and the region closer to the central domain selects the higher‐resolution simulation method. For instance, Jayaraman et al. combined all‐atom MD and coarse‐grained MD to study the influence of polyethylene glycol (PEG) architectures on the structural properties of water.^[46]^ Elliott et al. studied the absorption of the aqueous mono‐ and divalent salt electrolytes to fully polarizable charged graphene sheets by combining quantum mechanics and molecular mechanics (QM/MM).^[47]^ Giovannini et al. developed the QM/MM method for computing hyperfine coupling constants in an open shell molecular system.^[48]^ As shown in Figure 5, Jain et al. proposed a hybrid coarse‐grained MD/DPD/FEM multiscale modeling method to study the mechanical properties of polymer materials.^[49]^

#### Machine learning potential method

Machine learning has emerged as an indispensable catalyst for innovation in computational chemistry, seamlessly integrating the cost‐effectiveness of traditional empirical potentials with the precision of ab‐initio methods. By using machine learning potentials, researchers can access extensive temporal and spatial scales to gain profound insights into intricate molecular systems. Different from the traditional MD, the interaction among atoms is controlled by the neural network based on the relative distance matrix of atoms, and the neural network is obtained by sampling from the higher‐resolution simulations, such as the DFT and AIMD. For instance, Zhang et al. developed the DeePMD frame and studied the phase transition and provided a larger view mechanism of chemical reactions.[^50^, ^51^] Michaelides et al. proposed a precise model of the carbon atom to study the defect formation of graphene and the adsorption and diffusion of water.[^52^, ^53^]

### 2.4 Perspectives on the challenges and opportunities of the full‐scale modeling method

In brief, there are three main multi‐scale modeling approaches that have the potential to be developed into full‐scale modeling method. The onion method is the most direct and earliest developed multi‐scale modeling method, and the QM/MM is most widely used in the onion method. However, the QM/MM method is severely limited in its ability to describe chemical reactions within predetermined fixed areas and lacks the adaptability to keep up with changes over time and events. Moreover, the current calculation speed of this method is extremely slow, rendering it utterly unsuitable for conducting large space‐time scale simulations. Besides that, for coupling more modeling method with different scales, how to deal with the transition between different simulation methods is the key to determining the future development of the onion method.

The contradiction between simulation accuracy and the simulation space‐time size will accompany the development of the simulation method for a long time. In order to ease this conflict, one of the potential approaches is to give up the continuity of events and increase the sampling span of events. For instance, by combining KMC with DFT and MD, people can enhance the sampling efficiency of rare events that cannot be observed using the traditional DFT and MD method. However, to effectively use the KMC method, it is essential to have a basic understanding of the synthesis process. Currently, KMC is still powerless for the unknown complex synthesis system.

With the rapid development of AI technology, machine learning technology provides a new way of full‐scale modeling, which will hopefully resolve the contradiction between simulation accuracy and the simulation space‐time size. However, there are still limitations in its ability to achieve full‐scale simulation due to its inability to incorporate complex experimental conditions and capture intermediate evolution processes and states. Additionally, it heavily relies on the integrity of existing data. Overall, the current multiscale modeling approaches fall short in elucidating the complete synthesis mechanism of chemical experiments. Even so, the chemical simulation with machine learning technology shows great potential. We believe that the hybrid approach combining machine learning with the traditional multi‐scale modeling method is the most promising development direction for full‐scale modeling methods.

Clearly, there is still a considerable distance to cover in the development of full‐scale modeling techniques that enable us to perform large‐scale and rapid simulations for intricate chemical systems, attaining simulation scales and speeds comparable to those of molecular mechanics. Fortunately, the advancement of both hardware and software performance, coupled with the rapid development of theoretical methods, has enabled the further refinement of efficient full‐scale modeling techniques.

## 3 CONCLUSION

In this perspective, taking zeolite and polymer syntheses as examples, we introduce the characters and applications of various simulation methods with different resolution scales in chemical experiments. Recently, there have been significant advancements in computer performance, theoretical foundations, and supporting simulation software. However, as the examples show, regardless of the organic or inorganic systems, the synthesis mechanism always tends to span multiple resolution scales, and a single model cannot obtain the whole picture of the synthesis mechanism. Despite advancements in multiscale modeling methods, there is still a lack of complete process descriptions for chemical synthesis experiments through full‐scale simulation. Therefore, it is necessary to further develop the full‐scale modeling methods for chemical experiments. Apparently, theoretical computational chemistry is still faced with challenges in the mechanism research of chemical experiments. Fortunately, the advancement of software and hardware technology, coupled with the comprehensiveness of theoretical knowledge, makes it possible to construct a full‐scale simulation platform, which can help experimenters to improve the synthesis efficiency in the future.

## CONFLICT OF INTEREST STATEMENT

The authors declare no conflicts of interest.

## Data Availability

The authors confirm that the data supporting the views of this perspective are available within the article. Raw data that support the views of this perspective are available from the corresponding author upon reasonable request.
